# Erxiekang emplastra as adjunctive therapy for acute gastroenteritis in children: a single-center retrospective cohort study

**DOI:** 10.3389/fped.2026.1832504

**Published:** 2026-06-11

**Authors:** Cuiyun Fang, Yuan Zhou, Zhongli Jiang, Xiaoxue Su, Wei Fan

**Affiliations:** 1 Department of Nursing, Liyang People’s Hospital, Liyang, China; 2 Department of Pediatrics, Liyang People’s Hospital, Liyang, China

**Keywords:** acute gastroenteritis, children, herbal medicine, non-oral therapy, retrospective cohort study, transdermal drug delivery

## Abstract

**Background:**

Standard treatments for pediatric acute gastroenteritis (AGE) have critical limitations in symptom control and medication adherence among young children. This study evaluated the efficacy and safety of Erxiekang Emplastra as adjunctive therapy for AGE in children aged 1 to 14 years.

**Methods:**

This single-center retrospective cohort study reviewed electronic medical records of children hospitalized with AGE between January 2024 and March 2025. Eligible patients were categorized into an exposed group receiving Erxiekang Emplastra plus standard therapy and an unexposed group receiving standard therapy alone. The primary outcome was diarrhea duration; secondary outcomes included the 3-day Modified Vesikari Scale (MVS) and treatment-related adverse events. Multivariable linear regression was conducted to quantify the independent therapeutic benefits of the Erxiekang Emplastra.

**Results:**

A total of 1,089 eligible patients were included, with 417 in the exposed group and 672 in the unexposed group. The exposed group had significantly shorter diarrhea duration (3.83 vs. 4.92 days, *p* < 0.01) and lower 3-day MVS (2.90 vs. 4.51, *p* < 0.01). Adjunctive Erxiekang Emplastra was independently associated with reduced diarrhea duration (*β* = –0.38, *p* < 0.01; R^2^ = 0.19) and lower 3-day MVS (*β* = –0.44, *p* < 0.01; R^2^ = 0.22). Only 1.44% of patients developed mild transient local reactions, with no severe adverse events reported.

**Conclusion:**

Erxiekang Emplastra showed favorable tolerability in this pediatric AGE cohort, and its adjunctive use was independently associated with shortened diarrhea duration and reduced disease severity. Large-scale, multicenter, prospective randomized controlled trials are warranted to confirm the efficacy and safety of this intervention.

## 1 Introduction

Acute gastroenteritis (AGE) is a highly prevalent acute gastrointestinal disorder in the pediatric population, characterized by diffuse inflammatory injury of the gastric and intestinal mucosa, predominantly triggered by viral, bacterial, or parasitic pathogens (1, 2). It remains one of the leading causes of morbidity and mortality among children globally, particularly in low- and middle-income countries (3). It is estimated that AGE is responsible for approximately 525,000 annual deaths among children under 5 years of age worldwide; among these cases, life-threatening dehydration and electrolyte disturbances represent the most common severe complications and direct drivers of mortality (4). Beyond the acute health risks, AGE also exerts a substantial socioeconomic burden on healthcare systems and families, including increased healthcare resource utilization, direct medical costs, and indirect productivity losses secondary to parental work absenteeism (5, 6). The clinical guidelines jointly issued by the European Society for Paediatric Gastroenterology, Hepatology and Nutrition (ESPGHAN) and the European Society for Paediatric Infectious Diseases (ESPID) explicitly recommend oral rehydration therapy (ORT) and age-appropriate dietary management as the cornerstone of first-line treatment for pediatric AGE, with probiotics designated as an optional adjunctive intervention (7). However, oral-based therapeutic and rehydration interventions face substantial clinical challenges in the pediatric population, particularly in young children. Frequent and recurrent vomiting during the acute phase of AGE markedly impairs the gastrointestinal absorption of oral agents, while poor acceptability of and low treatment adherence to oral medications are highly prevalent in this age group (8). These well-recognized clinical barriers highlight a critical unmet need for non-oral, minimally invasive, and well-tolerated therapeutic strategies to improve clinical outcomes in children with AGE.

Erxiekang Emplastra, a herbal transdermal preparation approved by the National Medical Products Administration of China, has been widely used in clinical practice for the management of pediatric gastrointestinal disorders in China for decades. This delivery route enables sustained systemic absorption of active pharmaceutical ingredients while effectively bypassing the harsh gastrointestinal environment, avoiding hepatic first-pass metabolism, and overcoming the challenge of oral administration in pediatric patients (9). Erxiekang Emplastra is formulated with four standardized herbal extracts, namely *Syzygium aromaticum*, *Piper nigrum*, *Evodia rutaecarpa*, and *Cinnamomum cassia*, all of which have a long history of medicinal use for gastrointestinal ailments in Traditional Chinese Medicine. Mounting preclinical evidence has demonstrated that the bioactive components of these herbal extracts exert synergistic pharmacological effects, including anti-inflammatory activity, intestinal mucosal barrier protection, and regulation of gastrointestinal motility, all of which directly target the core pathophysiological hallmarks of AGE (10). These preclinical findings provide a mechanistic basis for the clinical application of Erxiekang Emplastra in pediatric AGE, but there remains a notable gap in translating these preclinical data into robust, standardized clinical evidence.

Despite its widespread clinical use in China, high-quality clinical evidence supporting the efficacy and safety of the Erxiekang Emplastra for pediatric AGE remains remarkably scarce. Studies using standardized, validated tools for disease severity assessment are particularly lacking. To address this critical evidence gap, we conducted a retrospective cohort study to systematically evaluate the efficacy and safety of the Erxiekang Emplastra as an adjunctive therapy to standard care in children aged 1 to 14 years with AGE. In this study, we applied the validated Modified Vesikari Scale (MVS) to standardize the assessment of AGE severity at baseline and during follow-up (11). Furthermore, we performed multivariable regression analyses to adjust for potential confounding factors and quantify the independent therapeutic efficacy of the Erxiekang Emplastra in this pediatric cohort. This study aimed to provide robust clinical evidence to fill the above gaps, and explore a feasible, well-tolerated non-oral adjunctive treatment option for pediatric AGE.

## 2 Materials and methods

### 2.1 Study design and ethical approval

This was a single-center, retrospective cohort study conducted at the Department of Pediatrics, Liyang People's Hospital, a tertiary care hospital in Jiangsu Province, China. All clinical treatments and relevant clinical outcomes of enrolled patients were finalized prior to the launch of this study, and all treatment decisions were made independently by attending physicians in routine clinical practice without any intervention or assignment from the research team. We retrospectively reviewed and analyzed electronic medical records of children hospitalized with AGE between January 2024 and March 2025. The study was performed in strict accordance with the ethical principles of the Declaration of Helsinki. The study protocol was approved by the Institutional Review Board of Liyang People's Hospital (protocol code: 2025029). Written informed consent from the patients’ guardians was waived due to the retrospective, non-interventional design of the study.

### 2.2 Study population

Patients were included if they met the following criteria: aged 1 to 14 years; met the diagnostic criteria for AGE (7), defined as ≥3 watery or mucus-like stools within a 24-hour period, with changes from baseline stool consistency and frequency, with or without vomiting, fever, or abdominal pain; complete medical records with follow-up until symptom resolution.

Patients were excluded if they had: comorbid chronic gastrointestinal disorders, including inflammatory bowel disease, congenital gastrointestinal malformations, or functional gastrointestinal disorders; immunodeficiency, malignancy, or severe organ dysfunction; incomplete medical records or loss to follow-up before symptom resolution; or receipt of other non-standard Traditional Chinese Medicine therapies during treatment.

This study adopted a retrospective real-world cohort design, with no pre-specified randomization or treatment allocation rules established before data collection, and all group assignments were performed exclusively through retrospective review of de-identified electronic medical records. For the purposes of this retrospective analysis, eligible patients were divided into two groups entirely according to the actual in-hospital treatment regimen they received during hospitalization. The exposed group received Erxiekang Emplastra combined with standard therapy, and the unexposed group received standard therapy alone. The decision of whether to prescribe adjunctive Erxiekang Emplastra was made solely by the patients’ attending pediatrician within our department, based on the child's individual clinical characteristics. Prior to finalizing the treatment plan, the attending physician fully informed the patients’ legal guardians of the patch's potential therapeutic benefits, standard application protocol, possible mild local adverse reactions, and associated medical costs. The final decision fully incorporated the guardian's treatment preferences, economic affordability, and willingness to receive this transdermal therapy. Throughout the study period, there were no mandatory indication restrictions, fixed medication quotas, or hospital administrative interventions governing the clinical use of Erxiekang Emplastra.

### 2.3 Standard therapy

Standard therapy for both groups followed ESPGHAN/ESPID guidelines (7): ORT with oral rehydration salts for dehydration prevention and correction; age-appropriate oral probiotics, specifically Saccharomyces boulardii or Bifidobacterium triple viable powder; symptomatic antipyretic treatment with ibuprofen or paracetamol for fever ≥ 38.5 °C; antibiotics only for patients with bacterial enteritis confirmed via stool culture and elevated inflammatory markers.

### 2.4 Erxiekang emplastra therapy

The exposed group received Erxiekang Emplastra in addition to the standard therapy described above. This herbal transdermal preparation is manufactured by Shanxi Jinxin Shuanghe Pharmaceutical Co., Ltd., with approval number Z20010126 granted by the National Medical Products Administration of China. To date, this preparation has not obtained marketing authorization or regulatory approval from any drug regulatory authority outside mainland China, with no official commercial supply in other countries or regions. The appearance of the patch used in this study is presented in Supplementary Figure S1. The patch was applied to a clean, dry umbilical area once daily for 6–8 h, for 3 to 5 consecutive days according to the patient's symptom resolution.

### 2.5 Data collection

Data were independently extracted from electronic medical records by two trained investigators. Any discrepancies between the two investigators were resolved through consensus discussion with a third senior investigator to ensure data accuracy and integrity. Extracted data covered core domains required for study analysis, including patient demographics, baseline clinical characteristics, laboratory parameters, treatment details, and predefined outcome data. Safety-related data, including all treatment-related adverse events (AEs) during the treatment period, were collected for the two groups.

### 2.6 Outcome measures

The primary outcome was duration of diarrhea, defined as the time from treatment initiation to diarrhea cessation. Diarrhea cessation was clearly predefined as a continuous 24-hour period with stool frequency < 3 times per day and stool consistency restored to the patient's normal baseline level. Secondary outcomes included the MVS after 3 days of treatment and the incidence of treatment-related AEs in the exposed group. The MVS, as shown in Table 1, is a validated scale for assessing the disease severity of pediatric AGE (11). It evaluates diarrhea and vomiting frequency and duration, fever severity, healthcare utilization, and treatment intensity, with higher scores indicating more severe disease. Collected AEs included local skin reactions such as erythema, pruritus, rash, and ulceration, as well as systemic adverse events, including allergic reactions.

**Table 1:** Modified vesikari score.

Points	0	1	2	3
Diarrhea duration (hours)	0	1–96	97–120	≥121
Maximum no. of diarrheal stools/24 h period (in the course of the disease)	0	1–3	4–5	≥6
Vomiting duration (hours)	0	1–24	25–48	≥49
Maximum no. of vomiting episodes/24 h period (in the course of the disease)	0	1	2–4	≥5
Maximum recorded fever	<37.0 °C	37.1–38.4 ℃	38.5–38.9 °C	≥ 39.0 °C
Future healthcare visit	0	N/A	Primary Care	Emergency department
Treatment	None	Rehydration with intravenous fluids	Hospitalization	N/A

N/A, not applicable.

### 2.7 Statistical analysis

Continuous variables were reported as mean ± SD or median (IQR), and categorical variables as frequencies (percentages). Group comparisons were performed using Student's t-test or Mann–Whitney U test for continuous variables and chi-square or Fisher's exact test for categorical variables. Multivariable linear regression was performed to quantify the independent therapeutic benefits of Erxiekang Emplastra on the two core outcomes: diarrhea duration and 3-day post-treatment MVS. Based on clinical relevance and previous evidence on influencing factors of pediatric acute gastroenteritis, we pre-specified the following covariates in the regression models: age, sex, pre-enrollment diarrhea duration, baseline MVS, presence of fever at admission, presence of vomiting at admission, inflammatory markers (white blood cell count, C-reactive protein), rotavirus infection status, and antibiotic use. All pre-specified covariates were simultaneously entered into the models to fully adjust for potential confounding, without stepwise variable selection. We also used a sensitivity analysis to test the robustness of the main findings. This analysis excluded all patients who received antibiotic treatment during hospitalization, and the same statistical methods as the main analysis were used to re-evaluate the primary and secondary efficacy outcomes. A two-tailed *p*-value < 0.05 was considered statistically significant. Statistical analysis was performed using SPSS (version 26.0; SPSS, Chicago, IL, USA).

## 3 Results

### 3.1 Patient enrollment

**Figure 1:**
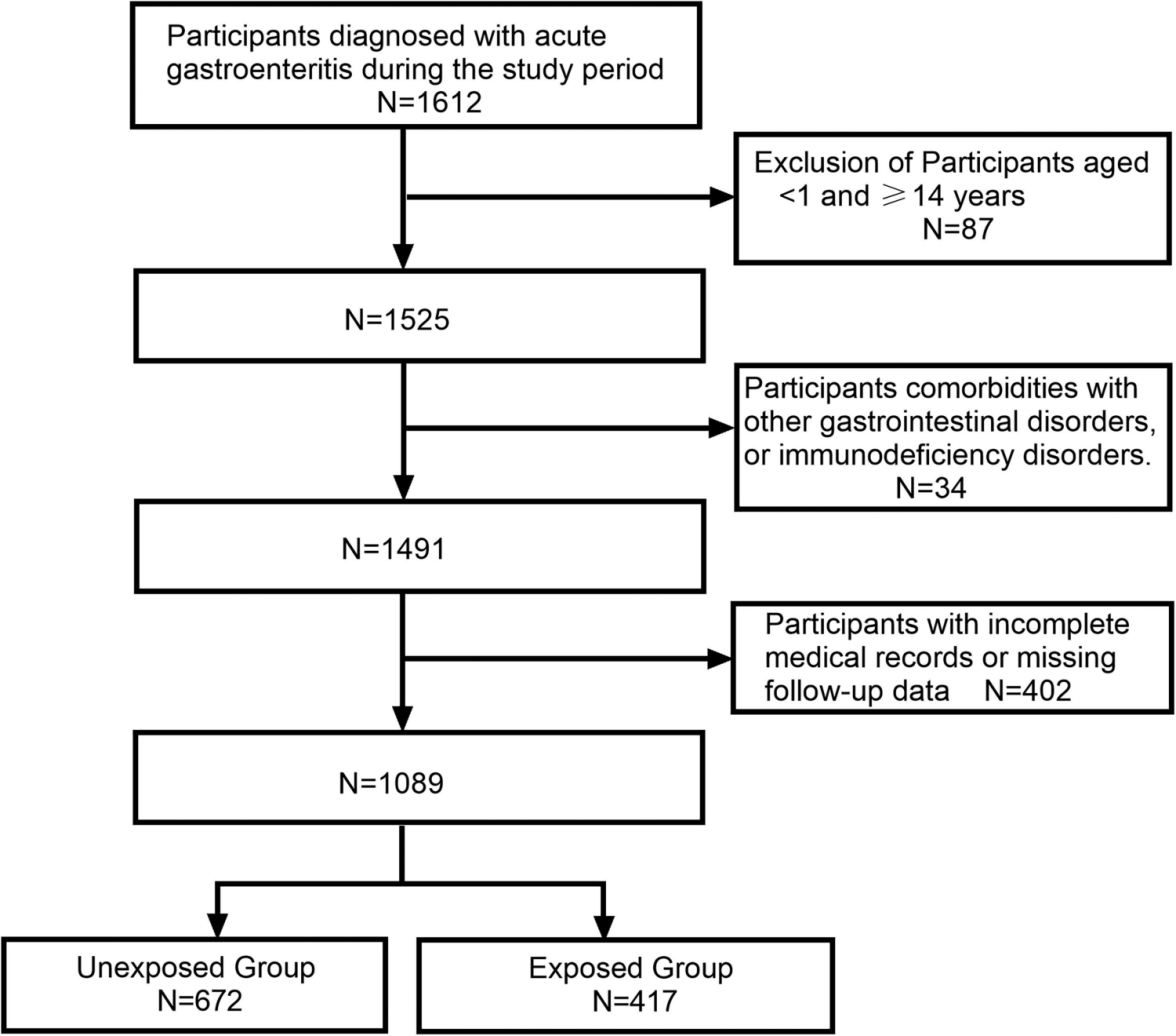
Patient selection flowchart for this retrospective cohort study of pediatric acute gastroenteritis.

As shown in Figure 1, a total of 1,612 pediatric patients with a primary discharge diagnosis of AGE were initially screened for eligibility between January 2024 and March 2025. Of these, 523 patients were excluded based on the predefined exclusion criteria: 87 were outside the eligible age range, 34 did not meet the standardized diagnostic criteria for AGE, and 402 had incomplete medical records or insufficient follow-up data. A total of 1089 eligible patients were ultimately included in the final analysis. Among them, 417 patients were allocated to the exposed group, and 672 patients were allocated to the unexposed group.

### 3.2 Baseline characteristics

The baseline demographic, clinical, and laboratory characteristics of the two groups are summarized in Table 2. There were no statistically significant differences between the exposed group and the unexposed group in age, sex, pre-enrollment diarrhea duration, baseline MVS, laboratory inflammatory markers, rotavirus positivity rate, presence of fever or vomiting at admission, or antibiotic use rate (all *p* > 0.05), confirming well-balanced baseline characteristics and high comparability between the two study groups.

**Table 2:** Comparison of baseline demographic and clinical characteristics between exposed and unexposed groups in children with acute gastroenteritis.

Characteristic	Unexposed Group (*N* = 672)	Exposed Group (*N* = 417)	*p* - value
Age (months)	66.91 ± 30.04	68.79 ± 38.36	0.37
Pre-enrollment diarrhea duration (hours)	24.82 ± 12.35	25.17 ± 13.06	0.66
Baseline MVS	6.31 ± 2.25	6.51 ± 2.28	0.16
Fever at admission, *n* (%)	328 (48.81%)	214 (51.32%)	0.42
Vomiting at admission, *n* (%)	402 (59.82%)	258 (61.87%)	0.50
White blood cells (×10^3^/µL)	9.20 ± 3.62	9.16 ± 3.46	0.88
Neutrophil (×10^3^/µL)	4.34 ± 3.03	4.24 ± 1.81	0.52
Lymphocyte (×10^3^/µL)	3.81 ± 2.10	4.10 ± 2.41	0.24
Platelets (×10^3^/µL)	295.12 ± 124.15	292.51 ± 120.90	0.73
C-reactive protein (mg/dL)	4.27 ± 6.75	3.39 ± 5.27	0.30
Sex, *n* (%)			0.48
Female	301 (44.79%)	196 (47.00%)	
Male	371 (55.21%)	221 (53.00%)	
Rotavirus, *n* (%)			0.16
Negative	91 (70.54%)	56 (61.54%)	
Positive	38 (29.46%)	35 (38.46%)	
Antibiotic use, *n* (%)	89 (13.24%)	52 (12.47%)	0.72

Data are presented as mean ± SD values/N (%).

N, number of patients; SD, standard deviation; MVS, Modified Vesikari Scale.

### 3.3 Primary and secondary efficacy outcomes

The primary and secondary efficacy outcomes of the two groups are summarized in Table 3. The exposed group had a significantly shorter mean duration of diarrhea compared with the unexposed group (3.83 ± 2.09 days vs. 4.92 ± 2.66 days; *p* < 0.01), corresponding to a mean 1.09-day reduction in diarrhea duration. After 3 days of treatment, the mean MVS was significantly lower in the exposed group than in the unexposed group (2.90 ± 2.01 vs. 4.51 ± 1.69; *p* < 0.01), demonstrating a significant reduction in disease severity with adjunctive Erxiekang Emplastra treatment.

**Table 3:** Comparison of 3-day modified vesikari score and diarrhea duration between exposed and unexposed groups.

Variables	Unexposed Group (*N* = 672)	Exposed Group (*N* = 417)	*P* - value
MVS after 3 days of treatment	4.51 ± 1.69	2.90 ± 2.01	<0.01
Duration of diarrhea(days)	4.92 ± 2.66	3.83 ± 2.09	<0.01

Data are presented as mean ± SD values.

N, number of patients; SD, standard deviation; MVS, Modified Vesikari Scale.

### 3.4 Multivariable linear regression analysis

Multivariable linear regression analysis was conducted to quantify the independent therapeutic effects of Erxiekang Emplastra on clinical outcomes, after adjusting for all pre-specified covariates, including age, sex, pre-enrollment diarrhea duration, baseline MVS, presence of fever at admission, presence of vomiting at admission, inflammatory markers, rotavirus infection status, and antibiotic use. As shown in Table 4, adjunctive treatment with Erxiekang Emplastra was independently associated with a significant reduction in diarrhea duration (*β* = –0.38, *p* < 0.01; R^2^ = 0.19). Erxiekang Emplastra use also conferred a significant independent benefit in reducing the 3-day post-treatment MVS (*β* = –0.44, *p* < 0.01; R^2^ = 0.22).

**Table 4:** Multivariable linear regression analysis of the independent effects of erxiekang emplastra on clinical outcomes.

Variables	Unstandardized Coefficients		Standardized Coefficients		*p*-value	R^2^	F	95% CI for B	
	B	SE	*β*	t				Lower bound	Upper bound
MVS after 3 days of treatment	−1.75	0.26	−0.44	−6.84	<0.01	0.22	6.04	−2.25	−1.25
Duration of diarrhea	−2.11	0.36	−0.38	−5.85	<0.01	0.19	4.98	−2.82	−1.40

CI, confidence interval; SE, standard error; MVS, Modified Vesikari Scale.

### 3.5 Sensitivity analysis

To evaluate the potential confounding effect of antibiotic use on clinical outcomes, a sensitivity analysis was conducted by excluding all patients who received antibiotic treatment during hospitalization. The efficacy outcomes and multivariable linear regression analysis were re-assessed using the same statistical methods as the main analysis. The detailed results are presented in Supplementary Tables S1, S2. Consistent with the main analysis, the exposed group still had significantly shorter diarrhea duration and lower 3-day MVS compared with the unexposed group (both *p* < 0.01). Multivariable linear regression analysis further confirmed that Erxiekang Emplastra use remained independently associated with improvements in both core clinical outcomes after adjustment for predefined confounding factors (both *p* < 0.01). These results were highly consistent with the main findings, confirming the stability and robustness of our core study outcomes.

### 3.6 Safety outcomes

Treatment-related AEs were retrospectively collected and systematically analyzed for the two groups. For the exposed group receiving additional adjunctive Erxiekang Emplastra, a total of 6 patients (1.44%) developed treatment-related mild local AEs during the treatment period, including 4 cases of periumbilical erythema and 2 cases of mild pruritus. All events were assessed as definitely related to Erxiekang Emplastra application, with a mild severity grade. None of these patients required additional pharmacologic management or treatment discontinuation due to the adverse events, and all symptoms resolved spontaneously within 24 to 48 h after patch removal. No systemic AEs associated with Erxiekang Emplastra were observed in the exposed group. No treatment-related AEs were observed in the unexposed group throughout the entire hospitalization period.

## 4 Discussion

In this single-center retrospective cohort study, we evaluated the efficacy and safety of Erxiekang Emplastra as adjunctive therapy for pediatric AGE in 1,089 children aged 1 to 14 years. First, the baseline analysis confirmed no statistically significant differences in demographic characteristics, baseline clinical features, laboratory parameters, and disease severity between the two groups (all *p* > 0.05). This well-balanced baseline laid a solid methodological foundation for the subsequent intergroup comparison of efficacy and safety, minimizing the interference of known clinically relevant confounding factors and ensuring the comparability of the two cohorts.

Based on the balanced baseline characteristics, our results demonstrated that adjunctive Erxiekang Emplastra, combined with guideline-directed standard therapy, was associated with a significant reduction in diarrhea duration and disease severity after 3 days of treatment, compared with standard therapy alone. This core finding has important clinical implications: shortening the duration of diarrhea not only rapidly relieves the cardinal symptoms of affected children, but also reduces the risk of dehydration, electrolyte imbalance and other secondary complications, providing an effective adjunctive therapeutic option for the clinical management of pediatric AGE.

Multivariable regression analysis further confirmed that Erxiekang Emplastra use was independently associated with improvements in both core clinical outcomes, after adjustment for predefined clinically relevant confounding factors. This analysis further strengthened the robustness of our core findings, excluding the interference of known confounding factors on the observed therapeutic association, and provided more rigorous statistical support for the clinical efficacy of Erxiekang Emplastra. In addition, the patch showed favorable safety and tolerability in this study cohort, with only a very low incidence of mild, transient local reactions reported in the exposed group. This safety profile is particularly valuable for the pediatric population: the non-oral transdermal administration route avoids potential gastrointestinal irritation caused by oral medications, and the extremely low incidence of AEs provides a reliable safety basis for its wide clinical application.

The observed clinical associations with improved outcomes in this study may be explained by two key features of the intervention: the synergistic pharmacological activities of its components targeting the core pathophysiological pathways of AGE, and a unique transdermal administration route that may be better suited for the pediatric population. Both features align with the known pathological hallmarks of pediatric AGE and the practical needs of clinical practice. The core pathological changes of pediatric AGE include intestinal mucosal inflammation, epithelial barrier injury, increased intestinal permeability, and dysregulated gastrointestinal motility, all of which are recognized as key contributors to diarrhea, vomiting, and other cardinal symptoms of the disease (12, 13). The four standardized herbal components of Erxiekang Emplastra have been shown in preclinical studies to act on these core pathological pathways. Specifically, the bioactive ingredients of *Evodia rutaecarpa* and *Syzygium aromaticum* have been reported to inhibit the Nuclear Factor-*κ*B (NF-*κ*B) and Mitogen-Activated Protein Kinase (MAPK) inflammatory signaling cascades, reduce the release of key pro-inflammatory cytokines including Tumor Necrosis Factor-alpha (TNF-α), Interleukin-1 beta (IL-1β), and Interleukin 6 (IL-6), alleviate intestinal mucosal inflammation, and protect intestinal epithelial barrier integrity in preclinical models (14–16). *Piper nigrum* and *Cinnamomum cassia* have also been found to further augment these anti-inflammatory and antioxidant effects, while regulating gastrointestinal smooth muscle motility, which may contribute to reduced diarrhea frequency (17, 18).

Beyond the potential pharmacological effects, the umbilical transdermal administration route of Erxiekang Emplastra may help overcome the core clinical challenges of oral interventions in pediatric AGE management. Poor adherence to oral medications is common in infants and young children, and frequent vomiting during the acute phase of AGE may lead to insufficient drug exposure and impaired oral bioavailability (19). Transumbilical administration bypasses the gastrointestinal mucosal barrier and avoids hepatic first-pass metabolism. Theoretically, it enables sustained and stable systemic absorption of active ingredients, which is particularly applicable to children with vomiting or poor oral tolerance. This non-invasive, easy-to-administer route may not only improve treatment acceptance in the pediatric population but also reduce the operational burden on caregivers and clinical staff, with potential practical value for both inpatient and outpatient care settings.

Compared with similar domestic and international studies, this study has significant methodological advantages and innovation value. Domestic previous clinical studies on Erxiekang Emplastra for pediatric AGE were mostly small-sample, single-arm observational studies, and most of them used subjective symptom relief rate as the primary outcome, lacking internationally validated standardized assessment tools, which limited the extrapolation and comparability of the research results. Internationally, existing studies on non-oral adjunctive therapy for pediatric AGE mostly focus on transdermal antiemetic preparations, and high-quality clinical evidence on herbal transdermal patches is extremely scarce (20). To our knowledge, this study includes one of the largest sample sizes to date for a clinical analysis of Erxiekang Emplastra for pediatric AGE, enrolling 1,089 eligible children aged 1 to 14 years. This sample size is larger than that of most previous similar studies, providing improved statistical power to detect the clinical effects of the therapy relative to prior small-scale investigations. Meanwhile, our study used the MVS, a widely validated tool globally, as the standardized instrument for disease severity assessment. This scale has been validated across diverse pediatric populations and clinical settings in Europe, North America, Africa, and Asia, which supports the standardization and objectivity of our outcome assessments and enables comparability of our findings with international peer studies (21, 22). In addition, our study employed multivariable linear regression analysis to estimate the independent association between Erxiekang Emplastra use and clinical outcomes, rather than merely identifying unadjusted associations. This approach provides a more precise estimate of the intervention's effect after accounting for key confounding factors. Furthermore, our study systematically reported the safety profile of the preparation in the exposed group, adding to the limited available clinical data on this formulation in the international academic literature. Our study fills this gap by providing large-sample clinical evidence for a non-oral adjunctive therapy and further quantifies its independent therapeutic benefit in reducing diarrhea duration. From a socioeconomic perspective, shortening the duration of diarrhea by an average of 1.09 days can reduce the length of hospital stay, decrease the risk of complications from prolonged diarrhea, and reduce parental work absenteeism, thereby lowering the overall medical and socioeconomic burden of pediatric AGE (23). The stability of our core findings was further validated by a sensitivity analysis excluding patients with antibiotic use. The results remained highly consistent with the main analysis, indicating that antibiotic use did not materially influence the observed therapeutic associations and confirming the robustness of our results.

Several limitations of this study must be acknowledged when interpreting the findings. First, this was a single-center retrospective cohort study, with group assignment performed exclusively through retrospective review of actual clinical treatment records rather than prospective randomization. Despite our rigorous adjustment for predefined clinically relevant confounding factors, and the well-balanced baseline characteristics between the two groups, there remains an inherent risk of unmeasured confounding and selection bias. Second, rotavirus antigen testing was only completed in approximately 20% of the included patients, meaning we were unable to fully adjust for the influence of different causative enteropathogens on disease course. Third, we did not conduct a long-term follow-up to evaluate the impact of the therapy on long-term intestinal function or the risk of recurrent AGE. Fourth, our findings have important limitations on generalizability. This single-center study was completed at a tertiary hospital in one Chinese city, with an exclusively ethnically Chinese cohort. This limits the applicability of our results to non-Asian populations, multi-ethnic cohorts, and other geographic regions. Furthermore, Erxiekang Emplastra is a Traditional Chinese Medicine transdermal preparation, with culturally specific clinical use and theoretical foundations. It is only approved for use in mainland China, with no regulatory approval or commercial supply outside the country. This further restricts the applicability of our findings to healthcare systems not focused on Traditional Chinese Medicine practice. Multicenter, large-scale, prospective randomized controlled trials are warranted to confirm the efficacy and safety of Erxiekang Emplastra, further elucidate its underlying molecular pharmacological mechanisms, and evaluate its long-term safety, cost-effectiveness, and applicability across diverse pediatric populations and care settings.

## 5 Conclusion

In this single-center retrospective cohort study of children with AGE, we found that adjunctive Erxiekang Emplastra combined with standard therapy significantly reduced diarrhea duration and disease severity vs. standard therapy alone. Multivariable regression confirmed that Erxiekang Emplastra use was independently associated with improved core clinical outcomes after adjusting for relevant confounding factors, and the preparation showed favorable safety and tolerability in the pediatric population with only mild transient local reactions and no severe treatment-related events. These findings indicate that Erxiekang Emplastra may serve as a promising non-oral adjunctive therapeutic option for pediatric AGE, addressing the clinical challenges of poor oral medication adherence and tolerance in the pediatric population. Given the inherent limitations of the single-center retrospective design of this study, multicenter, large-scale prospective randomized controlled trials are warranted to further confirm the efficacy, safety, and long-term clinical value of Erxiekang Emplastra in children with AGE.

## Data Availability

The original contributions presented in the study are included in the article/Supplementary Material, further inquiries can be directed to the corresponding author.
