# Supplementary material for: Erxiekang emplastra as adjunctive therapy for acute gastroenteritis in children: a single-center retrospective cohort study
Source: Front Pediatr. 2026 Jun 11;14:1832504. doi: 10.3389/fped.2026.1832504 (PMC13294084; doi:10.3389/fped.2026.1832504)
Supplement: Supplementary file 1 [file Supplementaryfile1.docx]

**TABLE S1 Sensitivity Analysis: Efficacy Outcomes After Excluding Patients Receiving Antibiotic Treatment**

| Variables | Unexposed Group (N=583) | Exposed Group (N=365) | *p*-value |
| --- | --- | --- | --- |
| MVS after 3 days of treatment | 4.56 ± 1.70 | 2.87 ± 2.01 | <0.01 |
| Duration of diarrhea(days) | 4.92 ± 2.66 | 3.80 ± 2.08 | <0.01 |

Data are presented as mean ± SD values.

N, number of patients; SD, standard deviation; MVS, modified Vesikari score.

**TABLE S2 Sensitivity Analysis: Multivariable Linear Regression Results After Excluding Patients Receiving Antibiotic Treatment**

| Variables | Unstandardized Coefficients | | Standardized Coefficients | | *p*-value | R² | F | 95% CI for B | |
| --- | --- | --- | --- | --- | --- | --- | --- | --- | --- |
|  | B | SE | β | t |  |  |  | Lower bound | Upper bound |
| MVS after 3 days of treatment | -1.77 | 0.28 | -0.44 | -6.30 | <0.01 | 0.23 | 5.27 | -2.32 | -1.22 |
| Duration of diarrhea | -2.21 | 0.39 | -0.40 | -5.63 | <0.01 | 0.20 | 4.44 | -2.98 | -1.43 |

CI, confidence interval; SE, standard error; MVS, modified Vesikari score.


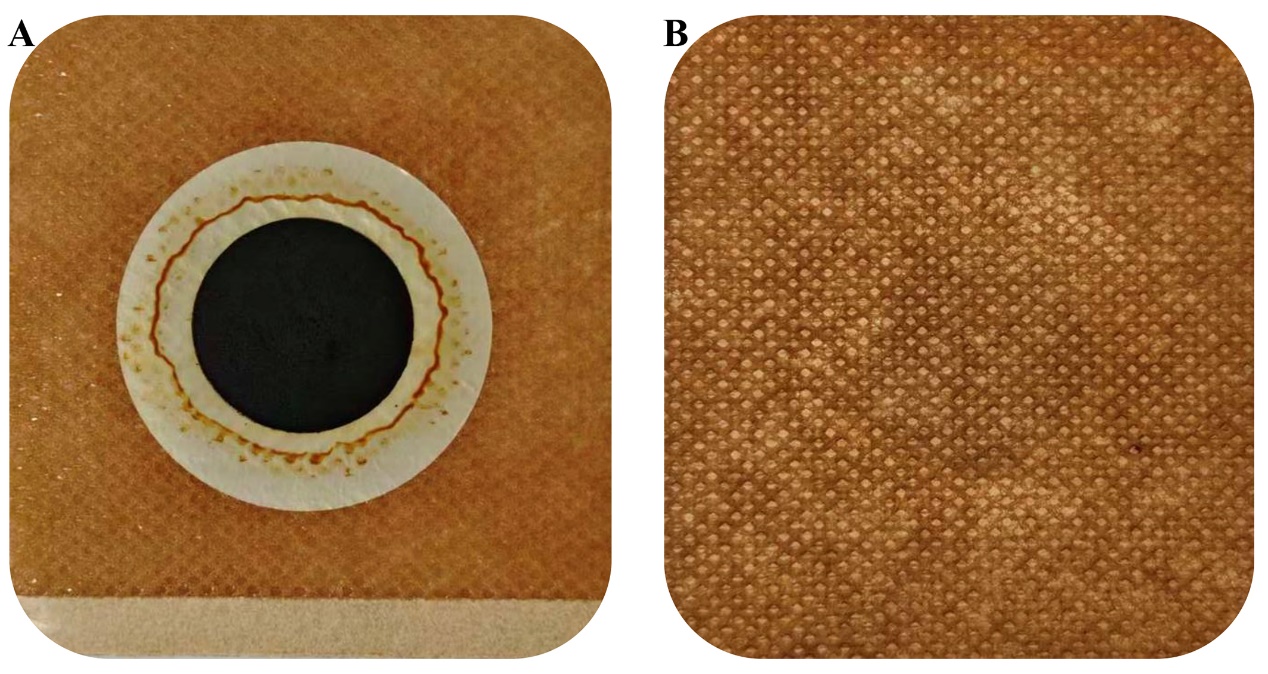
Figure S1. Appearance of Erxiekang Emplastra Used in This Study. (A) Front drug-loaded surface of the patch; (B) Back backing layer of the patch.
